# Long-Term Follow-Up Results of Adjuvant Intensity-Modulated Radiotherapy with Concurrent Paclitaxel and Cisplatin in High-Risk Endometrial Cancer Patients

**DOI:** 10.1155/2022/4621240

**Published:** 2022-10-11

**Authors:** Pei Shu, Xin Wang, Ganlu Ouyang, Jitao Zhou, Yaqin Zhao, Fang Wang, Zhiping Li, Yali Shen

**Affiliations:** Department of Abdominal Oncology, Cancer Center, West China Hospital, Sichuan University, No. 37 of Wainan Guoxue Lane, Chengdu, Sichuan Province, China 610041

## Abstract

**Purpose:**

The purpose of this study was to retrospectively review the outcomes of patients with high-risk endometrial cancer treated with adjuvant radiotherapy with concurrent paclitaxel and cisplatin (TP).

**Methods:**

Patients with endometrial cancer who underwent radical surgery were screened between Jan 2005 and Dec 2018. Patients with high-risk factors who received adjuvant chemoradiotherapy were included in the study. High risks included stage I, endometrioid-type grade 3 with deep myometrial invasion or lymphovascular space invasion (or both), endometrioid-type stage II to IVa, or stage I to III with serous or clear cell histology. The adjuvant treatment regimen included one cycle of TP chemotherapy, followed by pelvic intensity-modulated radiotherapy (IMRT) with concurrent TP, followed by an additional one cycle of TP. Failure free survival (FFS) and overall survival (OS) were estimated. Patterns of recurrence and occurrence of adverse events were described.

**Results:**

A total of 450 patients with high-risk endometrial cancer were screened, 231 of whom were included in this study. After a median follow-up of 70 months, the 5-year OS was 94.7%, and the 6-year OS was 91.8%. The 5-y and 6-y FFS were 90.8% and 87.9%, respectively, which were related to stage (*P* < 0.05). A total of 14 patients experienced tumor recurrence, including 7 pelvic recurrence and 7 distant metastases. Seven patients died, all due to tumor progression. A total of 164 patients (71%) completed the prescribed course of treatment. A total of 205 patients had adverse events, 46 patients (20%) had grade 1, 92 patients (40%) had grade 2, 49 patients (21%) had grade 3, and 18 patients (8%) had grade 4. There were 83 nonhematologic and 122 hematologic toxicities (26 grade 3 and 18 grade 4).

**Conclusion:**

Adjuvant pelvic radiotherapy combined with synchronous TP chemotherapy can achieve excellent long-term survival for high-risk endometrial cancer patients. Moreover, this combination therapy has good safety and feasibility, which is worthy of further study and verification.

## 1. Introduction

Endometrial carcinoma is the sixth most common cancer in women, with 417,000 new cases and 97,000 deaths in 2020 worldwide [1]. Approximately 15% of patients with endometrial cancer have high-risk features, and most have poor outcomes [2, 3]. The risk of disease progression was significantly higher in high-risk patients than in non-high-risk patients who also received surgical treatment (local recurrence (13% vs. 5%) and distant recurrence (19% vs. 3%)) [4]. Therefore, adjuvant therapy was considered.

The PORTEC-1 and GOG 99 trials showed that adjuvant EBRT significantly reduced the risk of vaginal and pelvic relapse compared with observation (14% vs. 4% in PORTEC1, *P* < 0.01; 13% vs. 5% in GOG99, *P* < 0.01) [4, 5]. Based on these trials, radiation therapy was recommended to patients with high-risk features. However, adjuvant radiotherapy fails to improve the overall survival. Approximately 20% to 30% distant failure rates for high-risk patients with observation were reported in the PORTEC-1 and GOG 99 trials [4, 5]. Adjuvant chemotherapy was considered appropriate to investigate. The comparison of adjuvant chemotherapy and pelvic EBRT was conducted in three randomized trials. The results showed that adjuvant chemotherapy reduced distant recurrence (16%-32% in chemotherapy versus 21%-38% in radiotherapy). The pelvic recurrence rate was lower in the radiotherapy group (18%-19% in the chemotherapy group versus 11%-13% in the radiotherapy group). Overall survival and relapse-free survival were similar between the two groups [6–8]. The complementarity of radiotherapy and chemotherapy is the basis for subsequent trials that focused on a combination of both in high-risk disease.

Five randomized clinical studies (Table 1) explored whether combination therapy could improve outcomes in high-risk endometrial cancer patients. In the pooled analysis of the NSGO 9501/EORTC 55991 trial and MaNGO ILIADE-III trial, progression-free survival was 7% higher in the chemoradiotherapy group than in the radiotherapy group (*P* = 0.009), but no significant difference was noted in overall survival [9]. In the PORTEC-3 trial, survival benefit of 5% overall survival and 7% relapse-free survival was shown in the chemoradiotherapy group compared with the radiotherapy group [10]. However, GOG-249 and GOG-258 trials did not show improved relapse-free survival or overall survival in the chemoradiotherapy group compared to chemotherapy alone [11, 12]. The inconsistencies in the results highlight the importance of identifying the optimal adjuvant treatment for high-risk endometrial cancer.

The purpose of this study was to provide an optional treatment method for high-risk endometrial cancer patients. A single institutions' experience using postoperative pelvic intensity-modulated radiotherapy (IMRT) with paclitaxel and cisplatin (TP) concurrent chemotherapy was reported in this study.

## 2. Methods

### 2.1. Ethical Considerations

The study was approved by the Ethics Committee of West China Hospital, Sichuan University, China (No. 2020-748).

### 2.2. Patient Selection and Eligibility Criteria

A retrospective review was conducted for women with high-risk endometrial cancer from 2005 to 2018 in West China Hospital, Sichuan University. The review was performed to identify all patients with high-risk endometrial cancer treated with radical surgery. The high-risk endometrial cancer was considered as International Federation of Obstetrics and Gynecology (FIGO) 2009 stage I, endometrioid-type grade 3 with deep myometrial invasion or lymphovascular space invasion (or both), endometrioid-type stage II to IVa, or stage I to III with serous or clear cell histology.

Patients with high-risk endometrial cancer who had received adjuvant chemoradiotherapy (radiotherapy with concurrent paclitaxel and cisplatin chemotherapy) were included. Patients were excluded if they received single-modality adjuvant therapy such as chemotherapy or radiation therapy only or neither.

### 2.3. Treatment

#### 2.3.1. Surgery

All patients had undergone total abdominal or laparoscopic hysterectomy with bilateral salping-oophorectomy and lymphadenectomy.

#### 2.3.2. Chemotherapy

Patients received one cycle of the TP regimen (paclitaxel 175 mg/m^2^, d1 and cisplatin 75 mg/m^2^, d1), followed by two cycles of the TP regimen with a decreased dose (paclitaxel 90 mg/m^2^, d1 and cisplatin 50 mg/m^2^, d1, q3w) during radiotherapy. After completion of chemoradiotherapy (CRT), patients received one additional cycle of chemotherapy with a standard TP regimen.

#### 2.3.3. Radiotherapy

Pelvic external-beam radiotherapy (EBRT) was given to patients after surgery. All patients were immobilized with abdominal body thermoplastic masks and treated in the supine position. Helical computed tomography at 3 mm slice thickness with intravenous contrast was performed for every patient. The clinical target volume (CTV) for radiotherapy was delineated according to the consensus guidelines for CTV delineation in postoperative pelvic radiation of endometrial and cervical cancer [13]. The clinical target volume included the upper 3 cm of the vagina, parametrial soft tissue, and pelvic regional lymph nodes (internal, external, and common iliac lymph nodes) up to the L5-S1 level. The clinical target volume was extended for lymph node involvement. A 0.6-0.8 cm uniform CTV expansion was applied to create the planning target volume (PTV).

A total dose of 50-50.4 Gy in 25-28 fractions was delivered. In patients with endometrioid-type grade 3 with both deep myometrial invasion and lymphovascular space invasion, an EBRT boost was given. A boost of 9 Gy/3 fractions was delivered to the upper two-thirds of the vagina, including the vaginal vault.

Plans were acceptable if the prescribed dose covered >95% of the PTV and no more than 1 cc received >107% of the prescribed dose. According to the Pelvic Normal Tissue Contouring Guidelines [14, 15], normal tissue constraints were as follows: less than 35% of the bladder to receive 50 Gy, less than 35% of the rectum to receive 50 Gy, less than 40% of the small bowel to receive 30 Gy, and less than 5% of the femoral heads to receive 50 Gy.

### 2.4. Follow-Up

Patients were followed up as scheduled: every 3 months for the first 2 years and every 6 months up to 5 years. Long-term outcome evaluation was obtained by follow-up visit. At each follow-up, a patient history, physical examination, and CA125 were performed. Radiologic assessments of chest and abdominal-pelvic were to be obtained every 6 months for the first 3 years and then annually for the next 2 years.

### 2.5. Outcomes

Analysis was performed to evaluate the effect of concurrent chemoradiotherapy. Overall survival (OS) was defined as the time from the date of surgery to the date of death from any cause. Failure-free survival (FFS) was defined as the interval between the date of surgery and the date of the first documentation of disease recurrence. Recurrences were analyzed according to the first site of recurrence.

Toxicity was assessed and graded with Common Terminology Criteria for Adverse Events (CTCAE) version 3.0.

### 2.6. Statistical Analysis

Descriptive statistics were used to quantify patient characteristics and toxicities. The Kaplan–Meier method was used to estimate overall and failure-free survival. Univariate and multivariate Cox regression analyses were performed to determine the influence of covariates on survival. Statistical significance was defined as *P* < 0.05. All statistical analyses were performed using SPSS version 22.0 (IBM Corp., Armonk, NY, USA).

## 3. Results

From January 2005 to December 2018, 450 patients with high-risk endometrial cancer were reviewed. Patients were excluded if they received single-modality adjuvant therapy such as chemotherapy or radiation therapy only or neither, or they did not receive postoperative radiotherapy with concurrent chemotherapy with paclitaxel and cisplatin (TP) (*n* = 219). A total of 231 patients were enrolled and analyzed in this study. The median follow-up time for patients was 70 months (IQR 48.1-90.3 months), and 144 patients (62.3%) had reached at least 5 years of follow-up. The median age was 55 years (range 27-81 years). All patients had >1 of the high-risk factors, and 80% of them had FIGO 2009 stage II-III disease.

All patients underwent hysterectomy and lymph node removal. The median number of pelvic lymph node (LN) dissections was 22 (3-45). Pelvic nodal and para-aortic nodal involvement were detected in 39 patients (16.6%) and 5 patients (2.1%), respectively. The majority of histology was endometrioid (86.1%). Among them, grades 2 and 3 were present in 156 patients (67.5%). Other types of histology included adenosquamous carcinoma (10.8%) and serous histology (2.2%). On histologic examination, 77% of patients had lymphovascular space invasion, 35.5% of patients had deep myometrial invasion, and 15.6% of patients had both of them above. The baseline characteristics of the patients are given in Table 2.

All patients received pelvic IMRT. Five patients received extra para-aortic lymph node radiotherapy in addition to pelvic radiotherapy. Otherwise, a boost of 9 Gy/3 fractions was delivered to 21 patients. A total of 98.3% of patients (227/231) completed planned-dose radiotherapy. Only 4 patients received an external beam pelvic radiotherapy dose of 44-46 Gy due to toxicity.

One hundred sixty-four patients (71%) completed all cycles of chemotherapy. Due to hematologic toxicity, 44 (19%) and 18 (8%) patients required a dose reduction of cisplatin and paclitaxel, respectively. During radiotherapy, 11 (5%) patients did not receive concurrent chemotherapy for toxicity. Four cycles of chemotherapy were given to 164 patients, and 3 cycles of chemotherapy were delivered to 63 patients.

**Figure 1:**
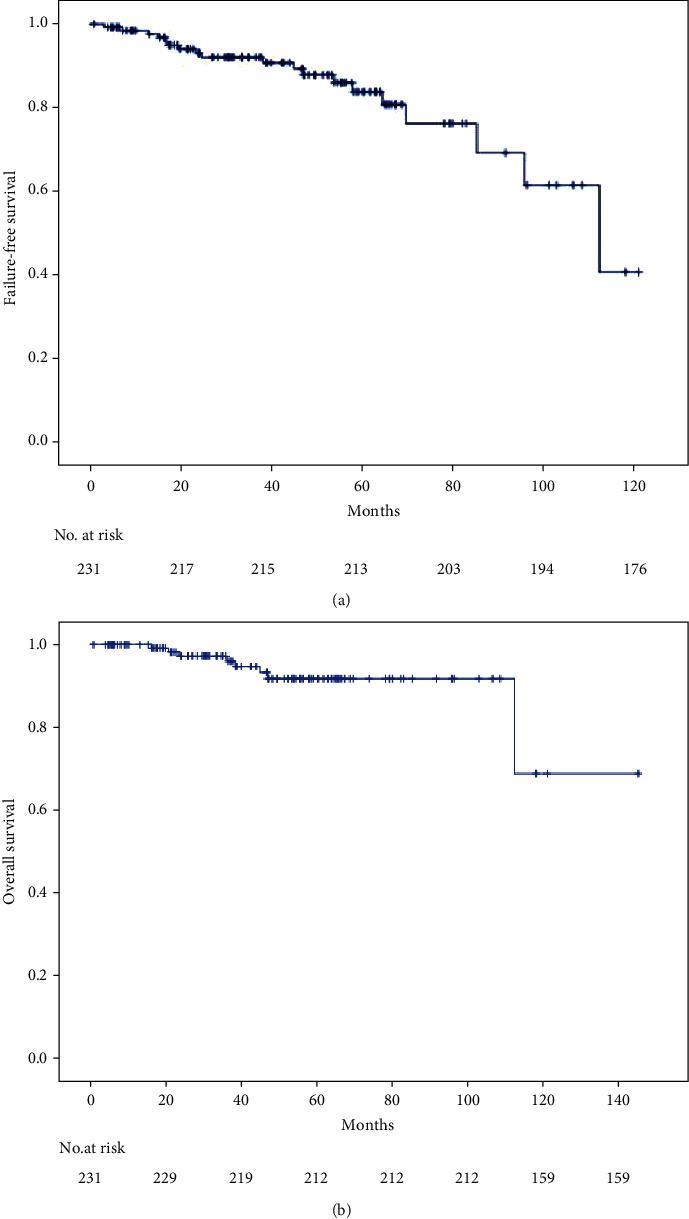
Kaplan-Meier survival curves for failure-free survival (a) and overall survival (b) in all patients.

The median overall survival was still not reached, nor was the median failure-free survival. In total, 7 deaths occurred during the whole follow-up period. All deaths were related to the progression of endometrial cancer. The 3 y, 5 y, and 6 y OS rates were 96%, 94.7%, and 91.8%, respectively. The 3-y, 5-y, and 6-y FFS rates were 93.1%, 90.8%, and 87.9%, respectively.  Figure 1 shows the OS and FFS curves.

Disease failure occurred in 14 (6%) patients. There were only 7 pelvic recurrences, which included 2 recurrences inside of the prior radiation field and 5 recurrences outside of the prior radiation field. The initial site of recurrence was extra-abdominal or hepatic in 6 patients. Only 1 patient had intrapelvic recurrence and synchronous distant metastasis together.

In univariate and multivariable analyses for OS and FFS, the following covariates were included: age, stage, histological type, grade, myometrial invasion, lymphovascular space invasion, and cervical junction involvement (Table 3). Univariate analysis showed that women with stage IIIC disease had much lower survival rates than those with stage I-IIIB disease. The five-year FFS and 5-year overall survival rates were 88.4% vs. 0% (HR 0·302, 95% CI 0·094–0.964; *P* = 0 · 043) and 97.4% versus 91.7% (HR 0.617, 95% CI 0·056–6.804; *P* = 0 · 693), respectively, for patients with different stages. In the multivariable analysis, none of the factors were significantly correlated with OS or FFS.

An overview of adverse events during and after treatment is provided in Table 4. Overall, adjuvant chemoradiotherapy was well tolerated. Most toxicities (60%) were grades 1-2. The rate of grade 3 or worse adverse events was reported to be 29%. During treatment, grade 3–4 acute adverse events were hematologic toxicities, which included grade 3-4 leukopenia or neutropenia in 35 patients and grade 3-4 anemia in 7 patients. Additionally, genitourinary (GU) or gastrointestinal (GI) adverse events were the second most common, occurring in 6 patients (2.6%) and 8 patients (3.5%), respectively. There was 1 patient with grade 3 liver damage recorded. There were no treatment-related deaths.

## 4. Discussion

To improve the prognosis of high-risk endometrial cancer (HREC), including local control and long-term survival, the role of adjuvant therapy needs to be further explored [16]. Phase III studies have shown that radiotherapy combined with chemotherapy can increase 5-y OS and FFS to 76.8%-85% and 59%-78% in high-risk endometrial cancer patients [11]. In this study, pelvic intensity-modulated radiotherapy (IMRT) combined with paclitaxel and cisplatin (TP) concurrent chemotherapy was applied, resulting in 5-y OS and FFS reaching 94.7% and 90.8%.

In different clinical trials, the specific implementation methods of radiotherapy and chemotherapy are different [9–12, 17, 18]. In the NSGO-EC-9501/EORTC-55991 trials, EBRT and four cycles of platinum-based chemotherapy given sequentially before or after EBRT were used [9]. In the clinical trial of PORTEC-3 and GOG 258, the treatment regimen was radiotherapy simultaneously with cisplatin, followed by paclitaxel and carboplatin for 4 cycles, which was the same as the RTOG-9708 trial [10, 11]. In the GOG 249 trial, vaginal brachytherapy followed by three cycles of carboplatin and paclitaxel was used [12]. In the RTOG 0921 trial, IMRT and concurrent cisplatin and bevacizumab followed by adjuvant carboplatin and paclitaxel for 4 cycles was used [17]. In this study, four cycles of TP chemotherapy were given, one cycle before radiotherapy, two cycles of TP chemotherapy simultaneously with pelvic IMRT, and one cycle of TP after radiotherapy. Different treatment regimens may bring differences in efficacy and side effects.

In the RTOG 0921 trial, adding bevacizumab to concurrent cisplatin-based chemoradiation increased the 2-year OS rate compared with previous study (97% vs. 90%) [17, 18]. Given this finding, it was postulated that the intensive concurrent treatment may further improve the outcome. Both cisplatin and paclitaxel were thought to have high activity in endometrial cancer and act as a radiation potentiator [19, 20]. The effects of adjuvant radiotherapy and concurrent cisplatin in high risk endometrial cancer have been revealed [11]. In addition, previous studies suggest that radiation with concurrent paclitaxel is well tolerated and effective for high-risk endometrial cancers [21, 22]. Paclitaxel plus platinum has been employed by Nomura et al. to evaluate the clinical benefit as postoperative adjuvant chemotherapy in endometrial cancer. The 5-year progression-free survival rate and 5-year overall survival rate were 73.9% and 86.1%, which were comparable with standard treatment [23]. Thus, the paclitaxel plus platinum regimen is an effective treatment for high-risk endometrial cancer. Given the impressive activity of paclitaxel and platinum in endometrial cancer and their radio-sensitizing properties, combination therapy of paclitaxel and cisplatin concurrent with radiotherapy is reasonable to explore [21, 24]. If TP regimen is given during radiotherapy, adverse events may increase with improved efficacy. Therefore, dose adjustment is the key when TP regimen is given simultaneously in radiotherapy. In this study, TP chemotherapy was given while in radiotherapy. The dose of the concurrent TP regimen was determined according to the tolerated dose obtained in the previous phase I study [25]. The tolerated dose of concurrent chemotherapy is paclitaxel 90 mg/m^2^ and cisplatin 50 mg/m^2^ [25].

As a precise irradiation technique, intensity-modulated radiotherapy (IMRT), compared with three-dimensional conformal radiotherapy (3D conformal radiotherapy), provide more accurate irradiation dose to the target region and better protection to adjacent normal organs. Reducing the irradiation range and dose to normal tissue can help reduce treatment-related toxicity [26, 27]. A study by Iğdem et al. showed a reduction in the volume of small bowel irradiated to more than 45 Gy with IMRT than with 4-field box radiation [28]. In the PORTEC-3 trial, which used 4-field conformal radiation, 14% of patients experienced grade 3 or 4 gastrointestinal toxicity [10]. In this study, the incidence of grade 3 and above gastrointestinal toxicity was 3.5% with IMRT.

Compared with other studies (listed in Table 1), the result of adverse events is acceptable in present study. In those phase III trials, the incidence of grade 3-4 toxicity was 51%-64.1%. In present study, the incidence of grade 3-4 toxicity was much lower, 29.1%. The combined scheme is safe and feasible, making the treatment completion rate in this study reach 71%, which is similar to that of PORTEC-3 [10]. Therefore, lower toxicity and better completion rate are important guarantees for the good long-term prognosis of this study. In this study, the patterns of treatment failure include 7 cases of local recurrence and 7 cases of distant recurrence. A total of 7 cases died due to disease progression during follow-up. In this study, the recurrence rate and mortality rate are low, indicating that the long-term treatment effect of high-risk endometrial patients is ideal. Given the small number of failure events, it is difficult to analyze the factors related to clinical outcome. Other studies have shown that staging is a prognostic factor for FFS, which is consistent with the conclusion of univariate analysis in this study [29].

Admittedly, this study has limitations. First, this is a retrospective study. Although more than 200 cases were included, there is still selection bias. In addition, this is a single-center study, and the treatment methods are relatively unified. It is impossible to compare the efficacy and safety with different radiotherapy and chemotherapy regimen. In the future, prospective clinical trials need to be carried out for research and verification. More trials should further address the use of concurrent treatment including chemotherapy or bevacizumab in patients with endometrial cancer.

## 5. Conclusions

This study showed that adjuvant pelvic radiotherapy combined with synchronous TP chemotherapy can achieve excellent long-term survival and good safety for high-risk endometrial cancer patients. It provides more clinical evidence for recommending radiotherapy and chemotherapy as the standard adjuvant treatment for high-risk endometrial cancer.

## Figures and Tables

**Table 1:** Summary of the main randomized controlled trials on adjuvant chemoradiotherapy for high-risk endometrial cancer.

Clinical trial	Number of patients	Treatment methods	Completion rate	LR	DM	5-year OS/DFS
PORTEC 3^10^	330	EBRT+ chemotherapy (consisting of two cycles of cisplatin 50 mg/m^2^ given during radiotherapy, followed by four cycles of carboplatin AUC 5 and paclitaxel 175 mg/m^2^)	71%	1.3%	22.4%	81.8%/75.5%
GOG 258^11^	346	EBRT + chemotherapy (consisting of two cycles of cisplatin 50 mg/m^2^ given during radiotherapy, followed by four cycles of carboplatin AUC 5 and paclitaxel 175 mg/m^2^)	75%	13%	27%	76.8%/59%
NSGO/EORTC pooled with Iliade-III^9^	267	EBRT+ chemotherapy (consisting of four cycles of AP or EP or TAC or TEC or TC)	72%	1%	6.6%	82%/78%
GOG 249^12^	300	VBT +chemotherapy (consisting of three cycles of carboplatin AUC 6 and paclitaxel 175 mg/m^2^)	87%	9%	18%	85%/76%

LR: local recurrence; DM: distant metastasis; OS: overall survival; DFS: disease-free survival; EBRT: external-beam radiotherapy; AP: doxorubicin 50 mg/m^2^ and cisplatin 50 mg/m^2^; EP: epirubicin 50 mg/m^2^ and cisplatin 50 mg/m^2^; TAC: paclitaxel 175 mg/m^2^ and doxorubicin 40 mg/m^2^ plus carboplatin AUC 5; TEC: paclitaxel 175 mg/m^2^ and epirubicin 50 mg/m^2^ and carboplatin AUC 5; TC: paclitaxel 175 mg/m^2^ and carboplatin AUC 5-6; VBT: vaginal brachytherapy.

**Table 2:** Characteristics of patients (*n* = 231).

Variables	No. of patients (%)
Age (years), median (range)	55 (27-81)
FIGO 2009 stage	
Stage IA	9 (4)
Stage IB	33 (14.3)
Stage II	97 (42)
Stage IIIa	33 (14.3)
Stage IIIb	12 (5.2)
Stage IIIc	43 (18.6)
Stage IV	4 (1.7)
Histological grade and type	
EEC grade 1	43 (18.6)
EEC grade 2	73 (31.6)
EEC grade3	83 (35.9)
Serous	5 (2.2)
Adenosquamous cell carcinoma	25 (10.8)
Clear cell carcinoma	1 (0.4)
Neuroendocrine carcinoma	1 (0.4)
Myometrial invasion	
<50%	63 (27.3)
>50%	82 (35.5)
Missing	86 (37.2)
LVSI	
Yes	179 (77.5)
No	5 (2.2)
Unknown	47 (20.3)
Lymphode positive	39 (16.9)
Parametrium invasion	9 (3.9)

FIGO: International Federation of Obstetrics and Gynecology; EEC: endometrial endometrioid cancer; LVSI: lymph-vascular space invasion.

**Table 3:** Univariate prognostic factor analysis.

Factors	*N*	3 y-FFS (%)	*P* ^∗^	3 y-0S (%)	*P* ^∗^
Age (years)					
<60	156	90.5	0.726	95.1	0.812
60-69	58	96.4		95	
≥70	17	90.9		90	
T-category					
≤T2	149	92.3	0.05	100	0.497
>T3	82	64.3		80	
N-category					
N+	39	94.1	0.465	100	0.054
N-	192	75.9		89.1	
Stage					
I-IIIB	184	88.4	0.043	97.4	0.693
IIIC-IV	47	0		91.7	
Tumor grade					
G1-2	116	90	0.505	95.8	0.054
G3	115	90.6		100	
Myometrial invasion					
<50%	63	93.6	0.652	100	0.221
>50%	82	91.4		94.9	
Parametrium invasion					
Yes	9	87.4	0.947	96.2	0.532
No	107	100		100	
Cervical junction involvement					
Yes	96	92.2	0.384	98	0.055
No	39	85.2		88.2	

FFS: failure-free survival; OS: overall survival.

**Table 4:** Grade 3-4 acute toxicity.

AE	Grade 3 *N* (%)	Grade 4 *N* (%)
Gastrointestinal toxicity	8 (3.5%)	0
Hematologic toxicity		
Hemoglobin	3 (1%)	4 (2%)
Leukocyte	21 (9%)	14 (6%)
Platelet	2 (1%)	0
Diarrhea	3 (1%)	0
Fatigue	5 (2%)	0
Genitourinary	6 (2.6%)	0
Liver function	1 (1%)	0

AE: adverse event.

## Data Availability

The datasets analyzed during the current study can be obtained from the corresponding author on reasonable requirements.

## References

[1] Sung H., Ferlay J., Siegel R. L. (2021). Global cancer statistics 2020: GLOBOCAN estimates of incidence and mortality worldwide for 36 cancers in 185 countries. *CA: a Cancer Journal for Clinicians*.

[2] Creutzberg C. L., van Putten W., Wárlám-Rodenhuis C. C. (2004). Outcome of high-risk stage IC, grade 3, compared with stage I endometrial carcinoma patients: the postoperative radiation therapy in endometrial carcinoma trial. *Journal of Clinical Oncology*.

[3] Greven K. M., Randall M., Fanning J. (1990). Patterns of failure in patients with stage I, grade 3 carcinoma of the endometrium. *International Journal of Radiation Oncology • Biology • Physics*.

[4] Keys H. M., Roberts J. A., Brunetto V. L. (2004). A phase III trial of surgery with or without adjunctive external pelvic radiation therapy in intermediate risk endometrial adenocarcinoma: a gynecologic oncology group study. *Gynecologic Oncology*.

[5] Creutzberg C. L., van Putten W. L. J., Koper P. C. M. (2000). Surgery and postoperative radiotherapy versus surgery alone for patients with stage-1 endometrial carcinoma: multicentre randomised trial. *Lancet*.

[6] Maggi R., Lissoni A., Spina F. (2006). Adjuvant chemotherapy _vs_ radiotherapy in high-risk endometrial carcinoma: results of a randomised trial. *British Journal of Cancer*.

[7] Randall M. E., Filiaci V. L., Muss H. (2006). Randomized phase III trial of whole-abdominal irradiation versus doxorubicin and cisplatin chemotherapy in advanced endometrial carcinoma: a gynecologic oncology group study. *Journal of Clinical Oncology*.

[8] Susumu N., Sagae S., Udagawa Y. (2008). Randomized phase III trial of pelvic radiotherapy versus cisplatin-based combined chemotherapy in patients with intermediate- and high-risk endometrial cancer: a Japanese Gynecologic Oncology Group study. *Gynecologic Oncology*.

[9] Hogberg T., Signorelli M., de Oliveira C. F. (2010). Sequential adjuvant chemotherapy and radiotherapy in endometrial cancer - results from two randomised studies. *European Journal of Cancer*.

[10] de Boer S. M., Powell M. E., Mileshkin L. (2018). Adjuvant chemoradiotherapy versus radiotherapy alone for women with high-risk endometrial cancer (PORTEC-3): final results of an international, open-label, multicentre, randomised, phase 3 trial. *The Lancet Oncology*.

[11] Matei D., Filiaci V., Randall M. E. (2019). Adjuvant chemotherapy plus radiation for locally advanced endometrial cancer. *The New England Journal of Medicine*.

[12] Randall M. E., Filiaci V., McMeekin D. S. (2019). Phase III trial: adjuvant pelvic radiation therapy versus vaginal brachytherapy plus paclitaxel/carboplatin in high-intermediate and high-risk early stage endometrial cancer. *Journal of Clinical Oncology*.

[13] Small W., Mell L. K., Anderson P. (2008). Consensus guidelines for delineation of clinical target volume for intensity-modulated pelvic radiotherapy in postoperative treatment of endometrial and cervical cancer. *International Journal of Radiation Oncology • Biology • Physics*.

[14] Gay H. A., Barthold H. J., O’Meara E. (2012). Pelvic normal tissue contouring guidelines for radiation therapy: a radiation therapy oncology group consensus panel atlas. *International Journal of Radiation Oncology • Biology • Physics*.

[15] Emami B., Lyman J., Brown A. (1991). Tolerance of normal tissue to therapeutic irradiation. *International Journal of Radiation Oncology • Biology • Physics*.

[16] Albuquerque K., Chino J., Klopp A., Kamrava M., Beriwal S. (2018). Defining the place of adjuvant chemotherapy and radiation for high-risk endometrial cancer from recent randomized clinical trials: some answers, more questions. *International Journal of Radiation Oncology • Biology • Physics*.

[17] Viswanathan A. N., Moughan J., Miller B. E. (2015). NRG oncology/RTOG 0921: a phase 2 study of postoperative intensity-modulated radiotherapy with concurrent cisplatin and bevacizumab followed by carboplatin and paclitaxel for patients with endometrial cancer. *Cancer*.

[18] Milgrom S. A., Kollmeier M. A., Abu-Rustum N. R. (2013). Postoperative external beam radiation therapy and concurrent cisplatin followed by carboplatin/paclitaxel for stage III (FIGO 2009) endometrial cancer. *Gynecologic Oncology*.

[19] Ball H. G., Blessing J. A., Lentz S. S., Mutch D. G. (1996). A phase II trial of paclitaxel in patients with advanced or recurrent adenocarcinoma of the endometrium: a gynecologic oncology group study. *Gynecologic Oncology*.

[20] Choy H., Rodriguez F. F., Koester S., Hilsenbeck S., von Hoff D. D. (1993). Investigation of taxol as a potential radiation sensitizer. *Cancer*.

[21] Cho H., Nam B. H., Kim S. M. (2014). A phase 2 trial of radiation therapy with concurrent paclitaxel chemotherapy after surgery in patients with high-risk endometrial cancer: a Korean Gynecologic Oncologic Group study. *International Journal of Radiation Oncology • Biology • Physics*.

[22] Frigerio L., Mangili G., Aletti G. (2001). Concomitant radiotherapy and paclitaxel for high-risk endometrial cancer: first feasibility study. *Gynecologic Oncology*.

[23] Nomura H., Aoki D., Michimae H. (2019). Effect of taxane plus platinum regimens vs doxorubicin plus cisplatin as adjuvant chemotherapy for endometrial cancer at a high risk of progression: a randomized clinical trial. *JAMA Oncology*.

[24] Lincoln S., Blessing J. A., Lee R. B., Rocereto T. F. (2003). Activity of paclitaxel as second-line chemotherapy in endometrial carcinoma: a gynecologic oncology group study. *Gynecologic Oncology*.

[25] Shu P., Shen Y., Zhao Y. (2015). A phase I study of adjuvant intensity-modulated radiotherapy with concurrent paclitaxel and cisplatin for cervical cancer patients with high risk factors. *Medical Oncology*.

[26] Roeske J. C., Bonta D., Mell L. K., Lujan A. E., Mundt A. J. (2003). A dosimetric analysis of acute gastrointestinal toxicity in women receiving intensity-modulated whole-pelvic radiation therapy. *Radiotherapy and Oncology*.

[27] Mundt A. J., Mell L. K., Roeske J. C. (2003). Preliminary analysis of chronic gastrointestinal toxicity in gynecology patients treated with intensity-modulated whole pelvic radiation therapy. *International Journal of Radiation Oncology • Biology • Physics*.

[28] Ercan T., Alço G., Zengin F. (2009). Dosimetric comparison of intensity modulated pelvic radiotherapy with 3D conformal radiotherapy in patients with gynecologic malignancies. *European Journal of Gynaecological Oncology*.

[29] Homesley H. D., Filiaci V., Gibbons S. K. (2009). A randomized phase III trial in advanced endometrial carcinoma of surgery and volume directed radiation followed by cisplatin and doxorubicin with or without paclitaxel: a gynecologic oncology group study. *Gynecologic Oncology*.

